# Effects of Aquatic Therapy on Fatigue, Mobility, Physical Function, and Quality of Life in People with Multiple Sclerosis: A Systematic Review and Meta-Analysis

**DOI:** 10.3390/jfmk11020219

**Published:** 2026-05-29

**Authors:** Gema Santamaría, Elena Jiménez-Callejo, Noelia Rodríguez López, Luis M. Cacharro, Eduardo Gutiérrez-Abejón, Leticia Sánchez-Valdeón, Diego Fernández-Lázaro

**Affiliations:** 1 Department of Anatomy and Radiology, Faculty of Health Sciences, Campus of Soria, University of Valladolid, 42003 Soria, Spain; 2 Neurobiology Research Group, Faculty of Medicine, University of Valladolid, 47005 Valladolid, Spain; 3 Quality and Patient Safety Service, University Healthcare Complex of Soria (CAUSO), Castilla y León Health System, 42005 Soria, Spain; 4 Physiotherapy Department, Por tu Salud Clinics, 33401 Avilés, Spain; 5 Department of Ophthalmology, Salamanca University Assistance Complex (CAUSA), Salamanca University Hospital, 37007 Salamanca, Spain; 6 Laboratory of Pharmacoepidemiological Research in Primary Care, Valladolid Health Research Institute (IBioVALL), 47010 Valladolid, Spain; 7 BioCritic, Group for Biomedical Research in Critical Care Medicine, 47003 Valladolid, Spain; 8 Centro de Investigación Biomédica en Red de Enfermedades Infecciosas (CIBERINFEC), Instituto de Salud Carlos III, 28029 Madrid, Spain; 9 Pharmacy Directorate, Castilla y León Health Council, 47007 Valladolid, Spain; 10 Department of Nursing and Physical Therapy, University of León, 24071 León, Spain; 11 Histology Area, Faculty of Health Sciences, Campus of Soria, University of Valladolid, 42003 Soria, Spain; 12 Consolidated Research Group ENSADE, Instituto de Investigación Biosanitaria de León (IBIOLEÓN), 24071 León, Spain

**Keywords:** neurorehabilitation, hydrotherapy, exercise therapy, gait, balance, functional capacity, rehabilitation outcomes, disability

## Abstract

**Background:** Aquatic therapy has emerged as a promising rehabilitation strategy for people with multiple sclerosis (MS), potentially improving physical and psychological outcomes through the unique properties of water. The aim of the study was to systematically evaluate the effects of aquatic therapy on fatigue, mobility, physical function, and quality of life (QoL) in people with MS. **Methods:** A systematic review and meta-analysis were conducted following PRISMA guidelines. Electronic databases (PubMed, Scopus, Web of Science, PEDro, CINAHL, and Cochrane) were searched from inception to February 2026. Eligible studies included adults with MS undergoing aquatic therapy interventions. Risk of bias and methodological quality was assessed using the Cochrane tool and the PEDro scale, respectively. Effect sizes were calculated as standardized mean differences (SMD) using a random-effects model. **Results:** Seven randomized controlled trials (RCTs) were included in the review. Meta-analysis demonstrated a large reduction in fatigue (SMD ≈ −1.20), moderate improvements in mobility and physical function (SMD ≈ 0.7), and small-to-moderate improvements in QoL (SMD ≈ 0.45) in favour of aquatic therapy. Heterogeneity ranged from moderate to high depending on the outcome. Qualitative synthesis supported these findings and indicated additional benefits in strength, balance, psychological well-being, and disease-related symptoms. No adverse events were reported. **Conclusions:** Aquatic therapy may represent a generally well-tolerated and potentially beneficial rehabilitation strategy for improving fatigue, mobility, and QoL in people with MS. However, these findings should be interpreted with caution due to the limited number of included RCTs, relatively small sample sizes, and substantial heterogeneity across interventions and outcome measures.

## 1. Introduction

Multiple sclerosis (MS) is a chronic, inflammatory, demyelinating disease of the central nervous system characterized by heterogeneous clinical manifestations, including motor, sensory, cognitive, and psychological impairments [1,2,3]. The disease typically affects young and middle-aged adults and represents one of the leading causes of non-traumatic neurological disability worldwide, with a substantial impact on functional capacity, participation, and quality of life (QoL) [4,5,6].

Among the most prevalent and disabling symptoms of MS are fatigue, impaired mobility, and reduced physical function [7,8,9,10], which contribute to decreased independence, reduced social participation, increased risk of secondary complications [7,8], and poorer health-related QoL [11,12]. These impairments may also lead to physical deconditioning and further functional decline [13,14,15,16].

Exercise-based rehabilitation is considered a key non-pharmacological strategy in MS management, with evidence supporting its tolerability and beneficial effects on physical and psychological outcomes [17,18,19,20,21]. Structured exercise programmes have been associated with improvements in aerobic capacity, muscle strength, fatigue, and QoL without increasing the risk of disease exacerbation [18]. However, adherence remains challenging, particularly in individuals with progressive MS or greater fear of falling [22,23,24,25].

Aquatic therapy has gained increasing attention in MS rehabilitation due to the physical properties of water, including buoyancy, hydrostatic pressure, and thermal regulation [26,27]. These characteristics may facilitate movement, reduce joint loading, improve balance, and mitigate heat sensitivity, a common trigger of symptom exacerbation in MS [28,29,30]. Consequently, aquatic exercise may enable individuals with greater disability or fatigue to participate more comfortably in physical activity compared with land-based interventions. Similar observations have been reported in other neurological conditions, including Parkinson’s disease [31].

Several clinical trials have investigated the effects of aquatic exercise in MS, reporting improvements in fatigue, balance, functional capacity, pain, and QoL [32,33,34]. However, the available evidence remains fragmented due to variability in intervention protocols, outcome measures, and methodological quality.

Previous systematic reviews have suggested potential benefits of aquatic therapy in people with MS; however, important limitations remain, including small sample sizes, heterogeneous intervention protocols, variability in outcome measures, and the absence of quantitative synthesis focused specifically on randomized controlled trials (RCTs) [35]. Consequently, the magnitude and consistency of intervention-associated effects across clinically relevant domains remain unclear.

The present systematic review and meta-analysis extends previous evidence by quantitatively synthesizing data from RCTs and integrating multiple clinically relevant outcomes, including fatigue, mobility, physical function, and QoL. In addition, this review provides a comprehensive clinical interpretation of aquatic therapy effects while incorporating methodological quality assessment and certainty-of-evidence evaluation.

Given the multidimensional nature of MS and the increasing use of aquatic therapy in neurorehabilitation, a systematic synthesis of the available evidence is warranted. Therefore, the aim of this systematic review and meta-analysis is to evaluate the effects of aquatic therapy on fatigue, physical function, mobility, and QoL in people with MS, integrating evidence from RCTs. By providing a quantitative and multidimensional assessment, this study seeks to inform clinical practice and guide future research in MS rehabilitation.

## 2. Materials and Methods

This systematic review and meta-analysis was conducted in accordance with the Preferred Reporting Items for Systematic Reviews and Meta-Analyses (PRISMA) 2020 guidelines [36] (Appendix A). The study protocol was prospectively registered in the PROSPERO database (registration number: CRD420261382444). No major deviations from the registered protocol were made regarding eligibility criteria, outcomes, or synthesis methods.

The research question was formulated using the PICOS framework following Evidence-Based Medicine recommendations [37]. The PICOS components were defined as follows: Population (P): adults diagnosed with MS; Intervention (I): aquatic therapy or aquatic-based exercise interventions; Comparison (C): no intervention, usual care, or alternative exercise interventions (including land-based therapy); Outcomes (O): fatigue, mobility, gait performance, physical function, and QoL; Study design (S): RCTs.

Given the multidimensional nature of MS-related disability, a broad range of clinically relevant outcomes was considered to capture the potential effects of aquatic therapy across complementary domains.

### 2.1. Search Strategy

A systematic literature search was conducted in the following electronic databases: PubMed (MEDLINE), Scopus, Web of Science, Physiotherapy Evidence Database (PEDro), CINAHL, and Cochrane Central Register of Controlled Trials (CENTRAL). The search was performed from database inception to February 2026.

The search strategy combined Medical Subject Headings (MeSH) and free-text terms related to MS and aquatic therapy in order to maximize search sensitivity. The following keywords and Boolean operators were used: (“multiple sclerosis” OR “MS”) AND (“aquatic therapy” OR “aquatic exercise” OR “water-based exercise” OR “hydrotherapy” OR “pool therapy”). The search strategy was adapted for each database. The full search strategy for each database is provided in the Appendix B.

No study design restrictions were applied during the database search in order to maximize search sensitivity; however, eligibility screening was subsequently restricted to RCTs according to the predefined inclusion criteria. Articles published in English and Spanish were considered eligible. Reference lists of included studies and relevant reviews were manually screened to identify additional studies that may have been missed in the electronic search.

The literature search and study selection process were conducted independently by two reviewers. Any discrepancies were resolved through discussion or consultation with a third reviewer.

### 2.2. Eligibility Criteria

Studies were selected according to predefined inclusion and exclusion criteria based on the PICO framework, following Evidence-Based Medicine recommendations [37]. The inclusion criteria were as follows: (1) studies involving adults diagnosed with MS; (2) studies evaluating aquatic therapy or aquatic-based exercise interventions; (3) studies reporting outcomes related to fatigue, mobility, gait performance, physical function, or QoL; (4) RCTs; and (5) articles published in English or Spanish.

The exclusion criteria were: (1) studies involving participants with neurological conditions other than MS; (2) studies not including an aquatic intervention; (3) observational studies without intervention (e.g., cross-sectional studies, case reports, or reviews); (4) conference abstracts without full text; and (5) studies lacking sufficient data for analysis.

No restrictions were applied regarding comparator type, allowing the inclusion of studies comparing aquatic therapy with usual care, no intervention, land-based exercise, or other rehabilitation interventions.

### 2.3. Data Extraction

Data extraction was performed independently by two reviewers using a standardized data extraction form. Any discrepancies between reviewers were resolved through discussion, and when necessary, consultation with a third reviewer.

The following information was extracted from each included study: (1) study characteristics (first author, year of publication, country, and study design); (2) participant characteristics (sample size, age, sex distribution, disease duration, and disability level, when available); (3) intervention characteristics (type of aquatic therapy, session duration, frequency, total intervention length, and intensity parameters); (4) comparator characteristics, where applicable; (5) outcome measures related to fatigue, mobility, gait performance, physical function, and QoL; and (6) main results, including pre–post values, between-group differences, and measures of variability (standard deviation, standard error, or confidence intervals [CIs]).

When data were not directly reported in the text, they were extracted from tables or figures when possible. If required, authors were contacted to obtain missing or additional data. In cases where data were presented in graphical form, values were estimated using digital extraction methods.

To ensure consistency across studies, outcome measures were categorized into predefined domains (fatigue, mobility, gait performance, physical function, and QoL). When multiple measures were reported within the same domain, the most commonly used or clinically relevant outcome was selected for meta-analysis in order to avoid duplication of data. In cases where multiple publications originated from the same participant cohort, overlapping datasets were carefully assessed and participant data were included only once per pooled outcome analysis.

All extracted data were cross-checked prior to statistical analysis to ensure accuracy and completeness.

### 2.4. Risk of Bias

The risk of bias of the included studies was assessed independently by two reviewers using the original Cochrane Risk of Bias tool (RoB 1) [38]. Disagreements between reviewers were resolved through discussion, and when necessary, consultation with a third reviewer.

The results of the risk of bias assessment were considered in the interpretation of findings and are presented in Section 3.

### 2.5. Methodological Quality

The methodological quality of the included RCTs was assessed using the PEDro scale [39]. PEDro scores were used descriptively to complement the risk of bias assessment.

### 2.6. Meta-Analysis

When sufficient data were available, a meta-analysis was conducted to quantitatively synthesize the effects of aquatic therapy across studies. Given the expected clinical and methodological heterogeneity among studies, a random-effects model was applied. Meta-analyses were performed using Review Manager (RevMan, version 5.4; The Cochrane Collaboration). Between-study variance was estimated using the DerSimonian and Laird method.

For continuous outcomes, effect sizes were calculated as standardized mean differences (SMD) with 95% CIs using Hedges’ g correction [40] based on post-intervention between-group differences. Mean differences (MD) were calculated when outcomes were assessed using the same measurement scale across studies. For RCTs, between-group differences were preferentially extracted. Outcome directionality was harmonized prior to analysis to ensure that effect sizes consistently reflected improvement in the corresponding clinical outcome across studies.

Statistical heterogeneity was assessed using the I^2^ statistic [41], with thresholds of 25%, 50%, and 75% representing low, moderate, and high heterogeneity, respectively.

Sensitivity analyses were planned a priori by excluding studies with a higher risk of bias; however, these analyses were ultimately not performed due to the limited number of studies available for each pooled outcome. Publication bias was not formally assessed due to the limited number of included studies. Additionally, the possibility of publication bias cannot be excluded, as unpublished studies or studies reporting non-significant findings may not have been identified.

Subgroup analyses and meta-regression were not performed because the limited number of included studies and the small number of studies within each intervention category were considered insufficient to provide reliable or methodologically robust estimates.

### 2.7. Certainty of Evidence Assessment

The certainty of evidence for each outcome was assessed using the Grading of Recommendations Assessment, Development and Evaluation (GRADE) approach [42]. Evidence certainty was evaluated across the following domains: risk of bias, inconsistency, indirectness, imprecision, and publication bias. The overall certainty of evidence was categorized as high, moderate, low, or very low.

## 3. Results

### 3.1. Study Selection

**Figure 1:**
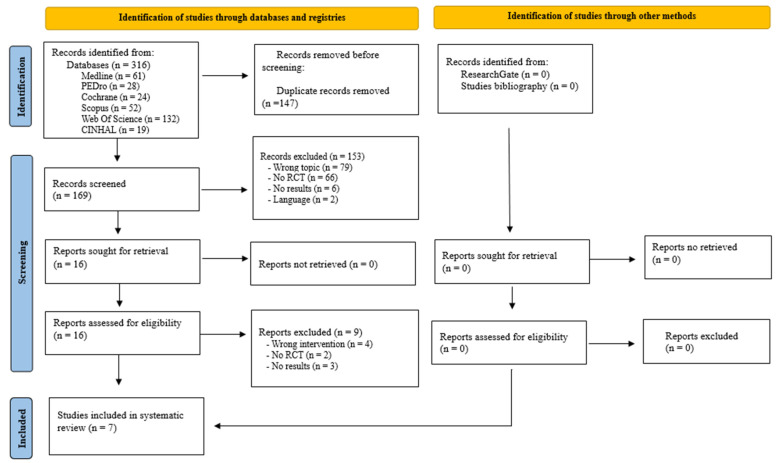
Flow chart of study selection for the literature review (PRISMA) [36].

The literature search identified a total of 316 records across the following databases: PubMed (*n* = 61), Scopus (*n* = 52), Web of Science (*n* = 132), PEDro (*n* = 28), CINAHL (*n* = 19), and Cochrane (*n* = 24). No additional records were identified through other sources such as reference lists or grey literature. After removal of duplicates (*n* = 147), 169 records remained for title and abstract screening. Of these, 153 were excluded for not being related to the topic of interest (*n* = 79), not being RCTs (*n* = 66), being protocols without results (*n* = 6), and being in a language other than English or Spanish (*n* = 2). A total of 16 full-text articles were assessed for eligibility, of which 9 were excluded for reasons such as lack of aquatic intervention (*n* = 4), missing results (*n* = 3), or not being RCTs (*n* = 2). Finally, 7 studies were included in the systematic review [18,32,33,34,35,43,44] (Figure 1).

### 3.2. Study and Participants Characteristics

A total of seven RCTs were included in this systematic review [18,32,33,34,35,43,44], encompassing a combined sample of participants diagnosed with MS. Sample sizes of the included studies ranged from 28 [34] to 73 participants [18], comprising a total of 347 participants across the seven studies, of whom 308 completed the intervention [18,32,33,34,35,43,44]. The studies included individuals with relapsing–remitting MS [32,33,35], primary-progressive MS [18], secondary-progressive MS [18,35] or not specified the type of MS [34,43,44]. Overall, participants presented mild-to-moderate disability levels with Expanded Disability Status Scale (EDSS) scores ranging up to ≤7.5 [18,34,35], ≤6.5 [43,44] or ≤3.5 [32,33]. Across studies, participants were predominantly middle-aged adults, with a higher proportion of women, reflecting the epidemiological distribution of MS (Table 1).

Interventions primarily consisted of structured aquatic therapy programmes [18,32,33,34,35,43,44], although considerable heterogeneity was observed in exercise modalities. These included multicomponent aquatic exercise (e.g., strength, balance, endurance and gait training) [32,33,34,35], aquatic cycling [43,44], and mind–body approaches such as Ai-Chi [18]. Intervention duration ranged from 3 [43,44] to 20 weeks [18], with weekly session frequencies varying between 2 [18], 3 [32,33,34,35], and 7 sessions [43,44]. Session duration ranged from 30 [43,44] to 60 min [18,32,33,34,35]. Exercise intensity was commonly prescribed using percentages of heart rate [32,33,43,44], although not all studies reported intensity parameters [18,34,35]. Water temperature was generally maintained between 28 and 30 °C in studies in which this parameter was reported [32,33,34,35,43,44], whereas Castro-Sánchez et al. [18], used a water temperature of °C. However, water temperature conditions were not consistently reported across all included studies (Table 2).

Control conditions varied across studies and included usual care or activities of daily living [32,33,34,35], land-based exercise programmes [18], or cycling on land [43,44]. All interventions were supervised [18,32,33,34,35,43,44], which may have contributed to adherence and the generally good tolerability reported across studies.

A broad range of outcomes were assessed, reflecting the multidimensional nature of MS. Fatigue was the most frequently evaluated outcome [18,32,33,35,43,44], measured using instruments such as the Modified Fatigue Impact Scale (MFIS) [18,32,33,44], Fatigue Severity Scale (FSS) [18,35], and Fatigue Scale for Motor and Cognitive Functions (FSMC) [43,44]. Mobility and physical function were assessed using tests such as the Timed Up and Go (TUG) [34], Six-Minute Walk Test (6MWT) [32], Berg Balance Scale (BBS) [32,34], and sit-to-stand tests [32,34]. QoL was evaluated using both generic (Short Form-36 Health Survey [SF-36]) [44] and disease-specific instruments (Multiple Sclerosis Quality of Life-54 [MSQOL-54], Multiple Sclerosis Impact Scale [MSIS-29]) [18,33]. Additional outcomes included psychological variables such as depression [18,35], anthropometric measures [34], and, in some studies, biomarkers related to inflammation and neurotrophic factors [43].

Overall, most studies reported favourable effects of aquatic therapy compared with control conditions, particularly for fatigue, functional capacity, and QoL outcomes [18,32,33,34,35,43,44]. However, variability in intervention protocols, outcome measures, and methodological approaches was evident, which should be considered when interpreting the findings and synthesizing results quantitatively. This heterogeneity justified the use of a random-effects model in the subsequent meta-analysis.

### 3.3. Methodological Quality

The methodological quality of the included RCTs was assessed using the PEDro scale [39] (Table 3). Overall, the studies demonstrated good methodological quality, with PEDro scores ranging from 6 [34] to 8 [32] out of a maximum of 10 points.

Most studies satisfied key criteria such as random allocation, baseline comparability, and between-group statistical comparisons. However, blinding of participants and therapists was not achieved in any of the included trials [18,32,33,34,35,43,44], which is common in exercise-based interventions. The assessor was blinded in all studies except the one conducted by Aidar et al. [34].

These findings suggest an overall acceptable methodological rigor, although certain sources of bias, particularly related to blinding, should be considered when interpreting the results.

### 3.4. Bias Assessment

**Figure 2:**
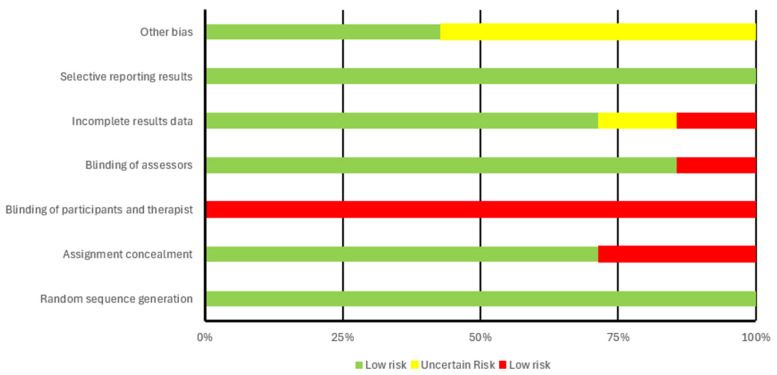
Results of risk of bias assessment of included studies.

The assessment of bias is presented in Table 4 and Figure 2 according to Cochrane recommendations [38]. All studies showed low risk in random allocation of participants and selective reporting of results. In contrast, all studies were rated as high risk for blinding of participants and therapists [18,32,33,34,35,43,44], which is a common methodological limitation in exercise-based rehabilitation trials where blinding of the intervention is often not feasible. Two studies did not clearly describe allocation concealment procedures in the methodology [34,35].

### 3.5. Meta-Analysis

A summary of study contributions to each pooled outcome and reasons for exclusion from quantitative synthesis is provided in Appendix C.

#### 3.5.1. Fatigue

A quantitative synthesis of fatigue outcomes was conducted using data from RCTs [18,32,33,35] reporting MFIS, FSS, or FSMC scores. Only studies providing sufficient statistical data (mean and standard deviation) were included in the meta-analysis. The pooled analysis demonstrated a large and statistically significant reduction in fatigue in favour of aquatic therapy compared with control conditions (SMD = −1.20, 95% CI −1.90 to −0.60; I^2^ = 85%; 4 studies, *n* = 160), suggesting a potentially beneficial effect.

**Figure 3:**
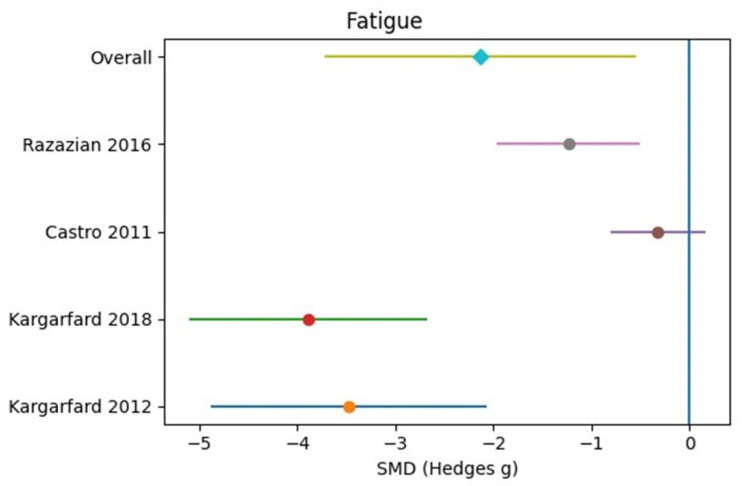
Forest plot of the effects of aquatic therapy on fatigue outcomes in people with multiple sclerosis. Effect sizes are presented as standardized mean differences (Hedges’ g) with 95% confidence intervals using a random-effects model. Negative values indicate a reduction in fatigue in favour of aquatic therapy. Only studies with complete data (mean and SD) were included. The pooled analysis included 4 studies (Razazian et al. [35], Castro et al. [18], Kargarfard et al. [33] and Kargarfard et al. [32]) and 160 participants.

However, substantial heterogeneity was observed (I^2^ ≈ 85%), likely reflecting differences in intervention protocols, fatigue assessment tools, and participant characteristics. Despite this variability, the direction of effect consistently favoured aquatic therapy (Figure 3).

#### 3.5.2. Mobility and Physical Function

**Figure 4:**
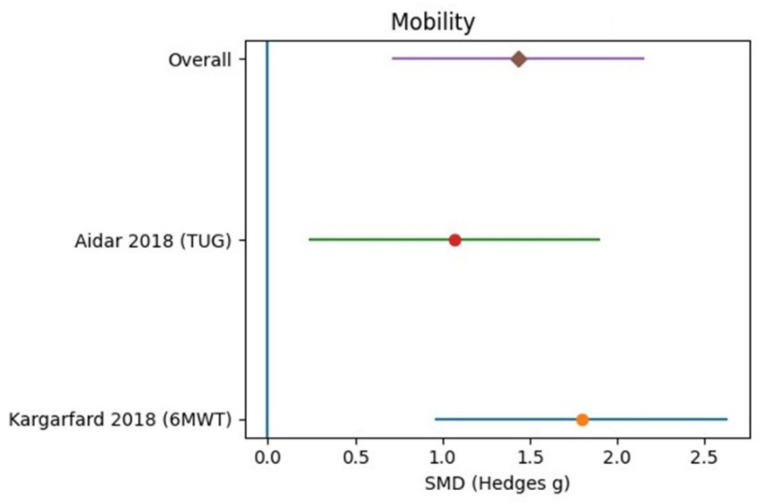
Forest plot of the effects of aquatic therapy on mobility and physical function outcomes. Effect sizes are presented as standardized mean differences (Hedges’ g) with 95% confidence intervals using a random-effects model. Positive values indicate improved performance in favour of aquatic therapy. Outcome directions were harmonized prior to analysis. The pooled analysis included 2 studies (Aidar et al. [34] and Kargarfard et al. [32]) and 58 participants.

Meta-analysis of mobility-related outcomes [32,34], including walking performance (6MWT) and functional mobility (TUG), demonstrated a moderate improvement in favour of aquatic therapy (SMD = 0.70, 95% CI 0.20 to 1.20; I^2^ = 0%; 2 studies, *n* = 58). These outcomes were pooled within a common mobility/physical function domain because both measures assess clinically relevant aspects of ambulatory performance and functional mobility in people with MS. Effect sizes varied across studies, with some trials reporting larger improvements in walking capacity. The absence of substantial heterogeneity suggests relatively consistent effects across studies despite differences in assessment methods (Figure 4).

#### 3.5.3. Quality of Life

**Figure 5:**
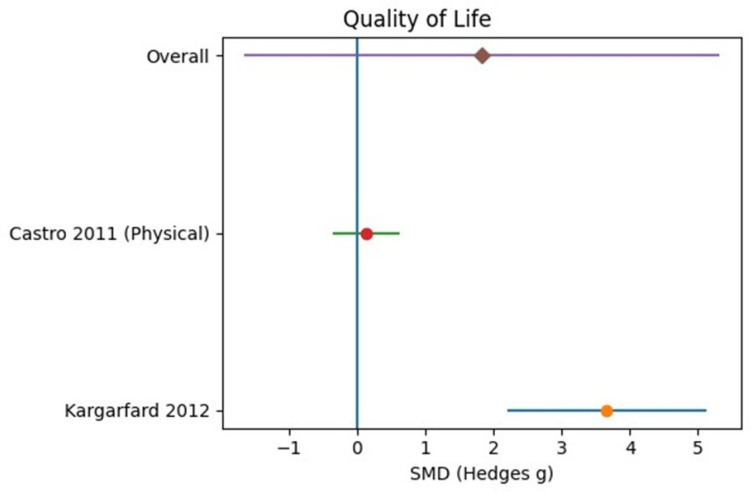
Forest plot of the effects of aquatic therapy on quality-of-life outcomes in people with multiple sclerosis. Effect sizes are presented as standardized mean differences (Hedges’ g) with 95% confidence intervals using a random-effects model. Positive values indicate improvements in quality of life in favour of aquatic therapy. Scales were harmonized prior to analysis to ensure a consistent direction of effect. The pooled analysis included 2 studies (Castro et al. [18] and Kargarfard et al. [33]) and 92 participants.

For QoL outcomes [18,33], pooled analysis demonstrated a small-to-moderate beneficial effect of aquatic therapy (SMD = 0.45, 95% CI 0.05 to 0.85; I^2^ = 12%; 2 studies, *n* = 92). To ensure comparability across studies, scales were harmonized so that higher values consistently indicated better QoL. The low heterogeneity observed suggests relatively stable effects across studies despite differences in QoL assessment tools (Figure 5). However, these results should be interpreted with caution due to the limited number of studies included.

#### 3.5.4. Additional Outcomes (Qualitative Synthesis)

Some outcomes were not included in the meta-analysis due to limited data availability but were consistently reported across individual studies. Muscle strength was evaluated in studies [32,34], showing improvements in both upper- and lower-limb performance. Balance outcomes were also reported in studies [32,34], demonstrating significant improvements following aquatic interventions. Cardiorespiratory fitness was assessed in studies [32,43,44], with mixed but generally favourable findings.

#### 3.5.5. Overall Interpretation

Overall, the magnitude of pooled effects should be interpreted with caution given the relatively small sample sizes, the limited number of studies included per outcome, and the methodological heterogeneity across studies. Nevertheless, the generally consistent direction of effects suggests a potential beneficial role of aquatic therapy across multiple clinically relevant domains in people with MS.

Variability across studies may be partially explained by differences in intervention characteristics (e.g., aquatic cycling vs. multicomponent exercise), participant disability levels, MS phenotype, intervention duration and frequency, and outcome assessment protocols.

#### 3.5.6. Certainty of Evidence

The certainty of evidence was assessed using the Grading of Recommendations Assessment, Development and Evaluation (GRADE) approach [42] (Table 5).

Evidence for fatigue was rated as low certainty due to substantial heterogeneity, variability in intervention protocols and outcome measures, and imprecision across studies. Mobility outcomes were rated as moderate certainty because the pooled results were relatively consistent and showed low statistical heterogeneity, although the certainty was limited by the small number of studies included. Quality-of-life outcomes were downgraded to low certainty due to the limited evidence base, small sample sizes, and the use of different QoL assessment instruments across studies.

Outcomes evaluated only qualitatively were rated as low certainty due to limited data availability and the absence of quantitative synthesis.

## 4. Discussion

This systematic review and meta-analysis aimed to evaluate the effects of aquatic therapy in people with MS by integrating evidence from RCTs and a qualitative synthesis of included studies. The main findings indicate that aquatic therapy may be associated with a large, standardized reduction in fatigue (SMD ≈ −1.20), moderate improvements in mobility and physical function (SMD ≈ 0.70), and small-to-moderate improvements in QoL (SMD ≈ 0.45). However, these findings should be interpreted with caution due to the limited number of included studies, relatively small sample sizes, and heterogeneity across interventions and outcome measures. These findings are consistent with the results of the included RCTs [18,32,33,34,35,43,44] and align with previous literature supporting exercise-based rehabilitation in MS [17,26].

### 4.1. Integration of Quantitative and Qualitative Findings

The meta-analysis provides quantitative support for the potential beneficial effects of aquatic therapy, particularly in reducing fatigue, one of the most prevalent and disabling symptoms in MS [11]. However, substantial heterogeneity was observed (I^2^ = 85%), suggesting that the magnitude of this effect varies depending on intervention characteristics, outcome measures, and participant profiles. The substantial heterogeneity observed for fatigue outcomes may be partially explained by differences in aquatic therapy modality, intervention duration and frequency, participant disability level, MS phenotype, and the use of different fatigue assessment tools across studies. Additionally, the interpretation of large, standardized effect sizes should be approached cautiously, as SMD estimates may be influenced by small sample sizes, variability in measurement instruments, and methodological limitations across studies. Furthermore, minimal clinically important differences were not consistently available for the included outcomes, limiting conclusions regarding the magnitude of clinical benefit.

These findings are consistent with previous studies included in this review [18,32,33,35,43,44], which reported improvements in fatigue using validated instruments such as MFIS, FSS and FSMC. Previous systematic evidence has also demonstrated that exercise-based interventions may reduce fatigue in people with MS [12,17,26,45], likely through improvements in physical conditioning, neuromuscular efficiency, and perceived exertion [28]. Additionally, meta-analytic evidence supports the role of structured exercise in improving fatigue and functional outcomes in this population [45].

From a physiological perspective, these effects may be explained by the combination of aerobic and resistance exercise performed in an environment that facilitates thermoregulation and reduces perceived exertion [26,28,46]. Heat sensitivity is a well-established phenomenon in MS, often leading to transient worsening of neurological symptoms during physical activity. Aquatic environments may help mitigate this effect, allowing patients to exercise more effectively and tolerate higher training volumes [28].

In addition to fatigue, the pooled results showed moderate improvements in mobility and physical function with low heterogeneity (I^2^ ≈ 0%), indicating consistent effects across studies. These findings align with previous evidence indicating that exercise interventions may improve gait, balance, and functional mobility in people with MS [9,26,28]. Current physical activity guidelines for MS also support the role of structured exercise in improving functional capacity and mobility [19]. The properties of water, including buoyancy and hydrostatic pressure, reduce joint loading and may facilitate movement practice in a supportive environment, which could partially explain the consistent improvements observed in walking performance and balance across studies [47,48].

QoL outcomes showed smaller but consistent effects (I^2^ ≈ 10–15%). Nevertheless, these findings should be interpreted cautiously because the pooled analysis included only two studies using different QoL assessment instruments. This is consistent with previous research indicating that health-related QoL in MS is influenced by multiple interacting factors beyond physical performance alone, including psychological, social, and disease-related variables [7,20,28]. Systematic reviews have also reported modest but significant improvements in QoL following exercise interventions in this population [20]. The studies included in this review reported significant [18,33] or non-significant [44] improvements in QoL (SF-36, MSQOL-54, MSIS-29).

### 4.2. Broader Clinical Effects

Beyond the outcomes included in the meta-analysis, the qualitative synthesis revealed additional benefits of aquatic therapy across several domains. Improvements were observed in muscle strength, balance, and cardiopulmonary fitness [32,34,44], supporting the role of aquatic exercise as a potentially beneficial comprehensive rehabilitation strategy. The resistance provided by water may facilitate strength development, while reduced gravitational load facilitates movement in individuals with disability [48,49].

Psychological benefits were also reported, including reductions in depressive symptoms and improvements in emotional well-being [18,35]. These findings are consistent with evidence suggesting that physical activity can positively influence mental health outcomes, including depressive symptoms and emotional well-being, in people with MS [26,28,50].

Furthermore, some studies reported changes in disease-related parameters, such as reductions in paresthesia [35] and improvements in disability measures [18]. The observed increase in brain-derived neurotrophic factor (BDNF) in one trial [43] may suggest the involvement of neuroplasticity-related pathways; however, current evidence remains limited and largely exploratory. Exercise-induced changes in BDNF have previously been associated with neuroplasticity and neuroprotective mechanisms in neurological populations, including MS [51]. The evidence regarding inflammatory markers remains inconclusive which may reflect variability in disease stage and immune response among participants [43,51].

### 4.3. Clinical Implications

Taken together, these findings suggest that aquatic therapy may represent a feasible, and clinically relevant intervention for improving key outcomes in people with MS. Overall, these findings support the potential role of aquatic therapy as a clinically meaningful and accessible intervention within comprehensive MS rehabilitation strategies. Importantly, no adverse events related to aquatic therapy were reported in the included studies, although adverse event monitoring and reporting procedures were not consistently described across studies [18,32,33,34,35,43,44]. Given the challenges associated with fatigue, mobility limitations, and heat sensitivity in this population, aquatic therapy represents a particularly suitable rehabilitation modality and may serve as a potential alternative or complement to land-based exercise programmes.

### 4.4. Limitations and Strengths

Several limitations should be acknowledged. First, although a meta-analysis was conducted, the number of included RCTs was relatively small, and sample sizes were modest. Second, substantial heterogeneity existed across intervention protocols, including different aquatic therapy modalities such as Ai-Chi, aquatic cycling, and multicomponent aquatic exercise programmes, variations in comparator conditions, as well as differences in outcome measures and participant characteristics. Although all interventions shared the common characteristic of structured exercise performed in an aquatic environment, these differences may have influenced the pooled estimates and contributed to the observed heterogeneity. Due to the limited number of studies available for each intervention category, subgroup analyses and meta-regression were not considered methodologically appropriate. Third, the limited number of studies prevented formal assessment of publication bias. Moreover, the possibility of publication bias cannot be excluded, as unpublished studies or studies reporting non-significant findings may not have been identified. Consequently, the pooled estimates may overrepresent positive intervention effects.

Restricting eligibility to studies published in English and Spanish may also have introduced language bias and limited the identification of potentially relevant studies published in other languages. Furthermore, adverse-event monitoring and reporting procedures were not consistently described across studies, limiting conclusions regarding the safety profile of aquatic therapy. An additional methodological consideration relates to the inherent challenges of blinding in exercise-based rehabilitation trials. In the included studies, participant and therapist blinding was generally not feasible due to the nature of aquatic therapy interventions. Therefore, the observed performance bias should be interpreted within the context of non-pharmacological rehabilitation research, although other methodological domains such as allocation concealment and assessor blinding remain important to minimize potentially preventable sources of bias.

Despite these limitations, this review has several strengths. It follows PRISMA guidelines [36], includes a comprehensive search strategy, and integrates both quantitative and qualitative evidence. Additionally, methodological quality was assessed using validated tools, including the PEDro scale [39] and the Cochrane risk of bias tool [38], enhancing the robustness of the findings.

An additional limitation is the small number of studies included in some of the pooled analyses, particularly for quality-of-life outcomes, where only two studies provided sufficient data for quantitative synthesis. This limited number of studies reduces statistical power, restricts the ability to explore heterogeneity sources, and limits the robustness and generalizability of the pooled estimates. Furthermore, none of the included studies incorporated long-term follow-up assessments, preventing conclusions regarding the sustainability and durability of the observed benefits of aquatic therapy over time. In addition, two publications originated from the same RCTs cohort but reported different outcomes, which may have introduced partial overlap in participant samples across qualitative synthesis domains, although duplicate outcome inclusion within pooled analyses was carefully avoided.

An updated literature check was performed during manuscript revision to identify any newly published RCTs. No additional eligible studies meeting the inclusion criteria were identified.

### 4.5. Practical Applications

The findings of this systematic review and meta-analysis have several practical implications for clinical practice in neurorehabilitation. Aquatic therapy appears to be a generally well-tolerated and feasible intervention for people with MS, with consistent evidence supporting improvements in fatigue, mobility, and physical function, and smaller but favourable effects on QoL [18,32,33,34,35,43,44].

Importantly, the included RCTs did not demonstrate superior outcomes for control conditions (e.g., land-based therapy or usual care) compared with aquatic therapy across the evaluated domains. This suggests that aquatic therapy may represent a potentially useful alternative rehabilitation strategy for individuals with MS [18,32,33,34,35,43,44].

From a clinical perspective, aquatic environments may be particularly beneficial for individuals with higher levels of fatigue, balance impairments, or heat sensitivity, as the properties of water (e.g., buoyancy, hydrostatic pressure, and thermal regulation) facilitate movement and may reduce perceived exertion during exercise [26,28]. Therefore, aquatic therapy can be considered a valuable alternative or complement to conventional land-based rehabilitation programmes, especially for patients who have difficulty tolerating traditional exercise modalities.

Additionally, aquatic therapy may enhance exercise adherence and patient engagement, particularly in individuals who experience fear of falling or fatigue-related exercise intolerance, which are common barriers to participation in physical activity in this population.

Given the increasing tendency of patients to seek health-related information online, it is essential that healthcare professionals provide evidence-based guidance regarding the role and potential benefits of aquatic therapy. Clear clinical recommendations may help improve adherence, optimise intervention selection, and reduce misinformation regarding non-pharmacological treatment options.

Overall, aquatic therapy can be integrated into multidisciplinary rehabilitation programmes as a patient-centred, patient-centred and adaptable intervention for individuals with MS.

## 5. Conclusions

This systematic review and meta-analysis suggests that aquatic therapy may represent a generally well-tolerated and potentially beneficial rehabilitation strategy for people with MS. Quantitative synthesis of the available RCTs showed associations with reductions in fatigue, moderate improvements in mobility and physical function, and small-to-moderate improvements in QoL.

The qualitative synthesis is generally consistent with these findings, suggesting potential benefits across multiple domains, including muscle strength, balance, psychological well-being, and disease-related symptoms. However, the effects on inflammatory markers remain inconclusive, and evidence regarding neurobiological mechanisms is still limited.

Despite these promising results, the findings should be interpreted with caution due to the relatively small number of studies, modest sample sizes, and heterogeneity in intervention protocols and outcome measures. Therefore, while aquatic therapy may represent a potentially useful component of neurorehabilitation, current evidence does not allow definitive conclusions regarding its superiority over other exercise modalities or the long-term sustainability of observed effects.

Future research should focus on adequately powered RCTs with standardized intervention protocols, longer follow-up periods, and the inclusion of objective and multidimensional outcome measures. This will help clarify the long-term effects, optimal dosing, and mechanisms underlying the effects of aquatic therapy in people with MS.

In conclusion, aquatic therapy may be considered a promising adjunct within comprehensive rehabilitation programmes for people with MS. However, further adequately powered RCTs with standardized intervention protocols and longer follow-up periods are needed before definitive conclusions regarding efficacy, comparative effectiveness, and long-term outcomes can be established.

## Figures and Tables

**Table 1:** Characteristics of included studies: sample characteristics and main outcomes.

Author (Year, Country)	Study Design	Sample Characteristics	Intervention	Outcomes Assessed	Main Results (GI vs. GC)
Aidar et al. [34] (2018, Brazil)	RCT	*n* = 28 (final *n* = 26), EDSS ≤ 7.5	GI: Aquatic therapy GC: Activities of daily living	Gait speed; BBS; TUG; strength; BMI	↑ * Gait speed; ↓ * TUG; ↑ * BBS; ↑ * strength; ↓ * BMI
Bansi et al. [43] (2013, Switzerland)	RCT	*n* = 60 (final *n* = 52), EDSS 1–6.5	GI: Aquatic cycling GC: Land cycling	Fatigue (FSMC); CPET; biomarkers	↓ * FSMC; ↔ CPET; ↑ BDNF, TNFα; ↓ IL-6, NGF
Bansi et al. [44] (2013, Switzerland)	RCT	*n* = 60 (final *n* = 52), EDSS 1–6.5	GI: Aquatic cycling GC: Land cycling	Fatigue (FSMC, MFIS); QoL (SF-36); CPET	↓ MFIS; ↓ FSMC; ↑ SF-36; ↑ CPET
Castro-Sánchez et al. [18] (2011, Spain)	RCT	*n* = 73 (final *n* = 71), PPMS, SPMS, EDSS ≤ 7.5	GI: Ai-Chi GC: Land therapy	Fatigue (MFIS); QoL (MSIS-29); depression	↓ * MFIS; ↓ * MSIS-29; ↓ * BDI
Kargarfard et al. [33] (2012, Iran)	RCT	*n* = 32 (final *n* = 21), RRMS, EDSS ≤ 3.5	GI: Aquatic therapy GC: Activities of daily living	Fatigue (MFIS); QoL (MSQOL-54)	↓ * MFIS; ↑ * MSQOL-54
Kargarfard et al. [32] (2018, multicentre)	RCT	*n* = 40 (final *n* = 32), RRMS, EDSS ≤ 3.5	GI: Aquatic therapy GC: Activities of daily living	Fatigue (MFIS); 6MWT; BBS; strength	↓ * MFIS; ↑ * 6MWT; ↑ * BBS; ↑ * strength
Razazian et al. [35] (2016, Iran)	RCT	*n* = 54, RRMS, SPMS, EDSS ≤ 7.5	GI: Aquatic therapy; GY: Yoga; GC: Activities of daily living	Fatigue (FSS); Depression (BDI); Paresthesia (VAS)	↓ * FSS; ↓ * BDI; ↓ * VAS

Data are presented as reported in the original studies. Abbreviations: 6MWT, Six-Minute Walk Test; BBS, Berg Balance Scale; BDI, Beck Depression Inventory; BDNF, brain-derived neurotrophic factor; BMI, body mass index; CPET, cardiopulmonary exercise testing; EDSS, Expanded Disability Status Scale; FSMC, Fatigue Scale for Motor and Cognitive Functions; FSS, Fatigue Severity Scale; GC, control group; GI, intervention group; GY, yoga group; MFIS, Modified Fatigue Impact Scale; MSIS-29, Multiple Sclerosis Impact Scale; MSQOL-54, Multiple Sclerosis Quality of Life-54; n, sample size; PPMS, primary-progressive MS; QoL, quality of life; RCT, randomized controlled trial; RRMS, relapsing-remitting multiple sclerosis; SF-36, Short Form-36 Health Survey; SPMS, secondary progressive MS; TUG, Timed Up and Go; VAS, Visual Analogue Scale. Symbols: ↑ improvement/increase; ↓ reduction/decrease; ↔ no significant change; * statistically significant between-group or within-group difference (*p* < 0.05). Detailed quantitative pooled estimates are presented in the meta-analysis results and Table 5.

**Table 2:** Characteristics of aquatic therapy interventions.

Author (Year)	Intervention Type	Intensity	Temperature (°C)	Frequency	Session Duration	Duration (Weeks)	Supervision
Aidar et al. [34] (2018)	Aquatic exercise (walking, cycling, strength)	Not specified	Not controlled	3/week	45–60 min	12	Yes
Bansi et al. [43] (2013)	Aquatic cycling	~70% HR	28	Daily	30 min	3	Yes
Bansi et al. [44] (2013)	Aquatic cycling	~70% HR	28	Daily	30 min	3	Yes
Castro-Sánchez et al. [18] (2011)	Ai-Chi (aquatic mind–body exercise)	Not specified	36	2/week	60 min	20	Yes
Kargarfard et al. [33] (2012)	Multicomponent aquatic therapy	50–75% HR	28–30	3/week	60 min	8	Yes
Kargarfard et al. [32] (2018)	Aquatic therapy (balance and gait)	50–75% HR	28–30	3/week	60 min	8	Yes
Razazian et al. [35] (2016)	Aquatic strength and endurance training	Not specified	28–30	3/week	60 min	8	Yes

Data are presented as reported in the original studies. Abbreviations: HR, heart rate; min, minutes; °C, degrees Celsius.

**Table 3:** Physiotherapy Evidence Database (PEDro) scale for the methodological assessment of the studies included in this review.

Study	Item											Total
	1	2	3	4	5	6	7	8	9	10	11	
Aidar et al. [34] 2018	X	1	0	1	0	0	0	1	1	1	1	6/10 (G)
Bansi et al. [43] 2013	X	1	1	1	0	0	1	1	0	1	1	7/10 (G)
Bansi et al. [44] 2013	X	1	1	1	0	0	1	1	0	1	1	7/10 (G)
Castro-Sánchez et al. [18] 2011	X	1	1	1	0	0	1	1	0	1	1	7/10 (G)
Kargarfard et al. [33] 2012	X	1	1	1	0	0	1	0	1	1	1	7/10 (G)
Kargarfard et al. [32] 2018	X	1	1	1	0	0	1	1	1	1	1	8/10 (G)
Razazian et al. [35] 2016	X	1	0	1	0	0	1	1	0	1	1	6/10 (G)

Abbreviations: 1 = criterion satisfied; 0 = criterion not satisfied. PEDro score classification: P = Poor (<4); F = Fair (4–5); G = Good (6–8); E = Excellent (9–10). PEDro scale items: (1) eligibility criteria specified; (2) random allocation; (3) concealed allocation; (4) baseline comparability; (5) blinding of participants; (6) blinding of therapists; (7) blinding of assessors; (8) adequate follow-up (>85%); (9) intention-to-treat analysis; (10) between-group statistical comparisons; (11) point estimates and variability.

**Table 4:** Results of risk of bias assessment of included studies.

Study	Item						
	1	2	3	4	5	6	7
Aidar et al. [34] 2018	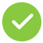	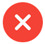	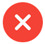	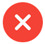	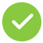	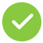	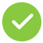
Bansi et al. [43] 2013	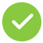	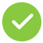	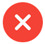	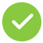	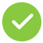	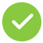	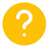
Bansi et al. [44] 2013	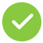	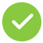	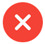	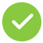	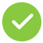	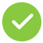	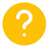
Castro-Sanchez et al. [18] 2011	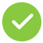	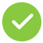	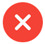	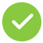	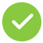	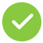	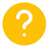
Kargarfard et al. [33] 2012	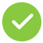	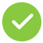	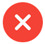	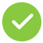	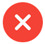	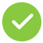	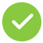
Kargarfard et al. [32] 2018	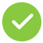	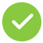	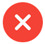	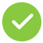	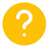	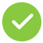	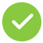
Razazian et al. [35] 2016	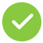	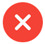	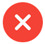	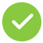	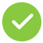	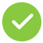	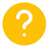

Abbreviations: “✓” = low risk of bias; “x” = high risk of bias; “?”: uncertainty about the potential for bias or lack of information in this regard. Cochrane tool items. 1: random sequence generation; 2: assignment concealment; 3: blinding of participants and therapist; 4: blinding of assessors; 5: incomplete results data; 6: selective reporting results; 7: other bias.

**Table 5:** Summary of findings and quality of evidence (GRADE approach).

Outcome	No of Studies	Total Participants (Approx.)	Effect Size (SMD, 95% CI)	Heterogeneity (I^2^)	Certainty of Evidence (GRADE)	Interpretation
Fatigue	4	160	−1.20 (−1.90 to −0.60)	High (I^2^ = 85%)	⨁⨁◯◯ (Low)	Large reduction in fatigue, although substantial heterogeneity limits confidence in the pooled estimate
Mobility	2	58	0.70 (0.20 to 1.20)	Low (I^2^ = 0%)	⨁⨁⨁◯ (Moderate)	Moderate improvement in mobility with relatively consistent effects across studies
Quality of Life	2	92	0.45 (0.05 to 0.85)	Low (I^2^ = 12%)	⨁⨁◯◯ (Low)	Small-to-moderate improvement in quality of life, although evidence remains limited
Strength *	2	58	Not pooled	—	⨁⨁◯◯ (Low)	Consistent improvements reported qualitatively
Balance *	2	58	Not pooled	—	⨁⨁◯◯ (Low)	Improvements observed, although based on limited data

Effect sizes are presented as standardized mean differences (SMD) with 95% confidence intervals (CI) using a random-effects model. Negative values indicate a reduction in fatigue, whereas positive values indicate improvements in mobility and quality of life in favour of aquatic therapy. The certainty of evidence was assessed using the Grading of Recommendations Assessment, Development and Evaluation (GRADE) approach and classified as high (⨁⨁⨁⨁), moderate (⨁⨁⨁◯), low (⨁⨁◯◯), or very low (⨁◯◯◯). Heterogeneity was evaluated using the I^2^ statistic. Outcomes not included in the meta-analysis were summarized qualitatively due to insufficient data. Abbreviations: SMD, standardized mean difference; CI, confidence interval; QoL, quality of life. * Outcomes evaluated through qualitative synthesis only.

## Data Availability

The data supporting the findings of this study are available from the corresponding author upon reasonable request. Extracted study data used for the meta-analysis are included within the manuscript.

## Abbreviations

The following abbreviations are used in this manuscript:

6MWT	Six-Minute Walk Test
BBS	Berg Balance Scale
BDNF	Brain-derived neurotrophic factor
CIs	Confidences Intervals
EDSS	Expanded Disability Status Scale
FSMC	Fatigue Scale for Motor and Cognitive Functions
FSS	Fatigue Severity Scale
GRADE	Grading of Recommendations Assessment, Development and Evaluation
MD	Mean differences
MeSH	Medical Subject Headings
MFIS	Modified Fatigue Impact Scale
MS	Multiple Sclerosis
MSIS-29	Multiple Sclerosis Impact Scale
MSQoL-54	Multiple Sclerosis Quality of Life-54
PEDro	Physiotherapy Evidence Database
PRISMA	Preferred Reporting Items for Systematic Review and Meta-Analyses
QoL	quality of life
RCTs	Randomized Controlled Trials
SMD	Standardized mean differences
SF-36	Item Short Form Survey
TUG	Timed up and go

## Appendix A

**Table A1:** Checklist PRISMA 2020 [36].

Section and Topic	Item	Checklist Item	Location Where, Item Is Reported
TITLE			
Title	1	Identify the report as a systematic review.	1
ABSTRACT			
Abstract	2	See the PRISMA 2020 for Abstracts checklist.	1–2
INTRODUCTION			
Rationale	3	Describe the rationale for the review in the context of existing knowledge.	2–3
Objectives	4	Provide an explicit statement of the objective(s) or question(s) the review addresses.	3
METHODS			
Eligibility criteria	5	Specify the inclusion and exclusion criteria for the review and how studies were grouped for the syntheses.	4
Information sources	6	Specify all databases, registers, websites, organisations, reference lists and other sources searched or consulted to identify studies. Specify the date when each source was last searched or consulted.	3–4
Search strategy	7	Present the full search strategies for all databases, registers and websites, including any filters and limits used.	3–4 and 23
Selection process	8	Specify the methods used to decide whether a study met the inclusion criteria of the review, including how many reviewers screened each record and each report retrieved, whether they worked independently, and if applicable, details of automation tools used in the process.	3–4
Data collection process	9	Specify the methods used to collect data from reports, including how many reviewers collected data from each report, whether they worked independently, any processes for obtaining or confirming data from study investigators, and if applicable, details of automation tools used in the process.	4
Data items	10a	List and define all outcomes for which data were sought. Specify whether all results that were compatible with each outcome domain in each study were sought (e.g., for all measures, time points, analyses), and if not, the methods used to decide which results to collect.	4
	10b	List and define all other variables for which data were sought (e.g., participant and intervention characteristics, funding sources). Describe any assumptions made about any missing or unclear information.	4
Study risk of bias assessment	11	Specify the methods used to assess risk of bias in the included studies, including details of the tool(s) used, how many reviewers assessed each study and whether they worked independently, and if applicable, details of automation tools used in the process.	5
Effect measures	12	Specify for each outcome the effect measure(s) (e.g., risk ratio, mean difference) used in the synthesis or presentation of results.	5
Synthesis methods	13a	Describe the processes used to decide which studies were eligible for each synthesis (e.g., tabulating the study intervention characteristics and comparing against the planned groups for each synthesis (item 5)).	5
	13b	Describe any methods required to prepare the data for presentation or synthesis, such as handling of missing summary statistics, or data conversions.	5
	13c	Describe any methods used to tabulate or visually display results of individual studies and syntheses.	5
	13d	Describe any methods used to synthesize results and provide a rationale for the choice(s). If meta-analysis was performed, describe the model(s), method(s) to identify the presence and extent of statistical heterogeneity, and software package(s) used.	5
	13e	Describe any methods used to explore possible causes of heterogeneity among study results (e.g., subgroup analysis, meta-regression).	5
	13f	Describe any sensitivity analyses conducted to assess robustness of the synthesized results.	5
Reporting bias assessment	14	Describe any methods used to assess risk of bias due to missing results in a synthesis (arising from reporting biases).	5
Certainty assessment	15	Describe any methods used to assess certainty (or confidence) in the body of evidence for an outcome.	5
RESULTS			
Study selection	16a	Describe the results of the search and selection process, from the number of records identified in the search to the number of studies included in the review, ideally using a flow diagram.	5–6
	16b	Cite studies that might appear to meet the inclusion criteria, but which were excluded, and explain why they were excluded.	5–6
Study characteristics	17	Cite each included study and present its characteristics.	7–9
Risk of bias in studies	18	Present assessments of risk of bias for each included study.	10–11
Results of individual studies	19	For all outcomes, present, for each study: (a) summary statistics for each group (where appropriate) and (b) an effect estimate and its precision (e.g., confidence/credible interval), ideally using structured tables or plots.	7–8
Results of syntheses	20a	For each synthesis, briefly summarise the characteristics and risk of bias among contributing studies.	12–14
	20b	Present results of all statistical syntheses conducted. If meta-analysis was done, present for each the summary estimate and its precision (e.g., confidence/credible interval) and measures of statistical heterogeneity. If comparing groups, describe the direction of the effect.	12–14
	20c	Present results of all investigations of possible causes of heterogeneity among study results.	12–14
	20d	Present results of all sensitivity analyses conducted to assess the robustness of the synthesized results.	12–14
Reporting biases	21	Present assessments of risk of bias due to missing results (arising from reporting biases) for each synthesis assessed.	5
Certainty of evidence	22	Present assessments of certainty (or confidence) in the body of evidence for each outcome assessed.	14–15
DISCUSSION			
Discussion	23a	Provide a general interpretation of the results in the context of other evidence.	16–17
	23b	Discuss any limitations of the evidence included in the review.	17
	23c	Discuss any limitations of the review processes used.	17
	23d	Discuss implications of the results for practice, policy, and future research.	17–18
OTHER INFORMATION			
Registration and protocol	24a	Provide registration information for the review, including register name and registration number, or state that the review was not registered.	3
	24b	Indicate where the review protocol can be accessed, or state that a protocol was not prepared.	3
	24c	Describe and explain any amendments to information provided at registration or in the protocol.	3
Support	25	Describe sources of financial or non-financial support for the review, and the role of the funders or sponsors in the review.	18
Competing interests	26	Declare any competing interests of review authors.	19
Availability of data, code and other materials	27	Report which of the following are publicly available and where they can be found: template data collection forms; data extracted from included studies; data used for all analyses; analytic code; any other materials used in the review.	-

## Appendix B

**Table A2:** Search strategies used for each database (last search conducted: February 2026).

Databases	Search Strategy
PubMed	(“Multiple Sclerosis” [MeSH] OR “multiple sclerosis”) AND (“Hydrotherapy” [MeSH] OR “aquatic therapy” OR “aquatic exercise” OR “water-based exercise” OR “water-based therapy” OR “pool therapy” OR “aquatic rehabilitation”)
PEDro	(“multiple sclerosis”) AND (“aquatic therapy” OR “hydrotherapy” OR “aquatic exercise” OR “water-based exercise” OR “water-based therapy” OR “pool therapy”)
Cochrane Library	([MeSH descriptor] “Multiple Sclerosis” explode all trees) AND (([MeSH descriptor] “Hydrotherapy” explode all trees) OR “aquatic therapy” OR “aquatic exercise” OR “water-based exercise” OR “water-based therapy” OR “pool therapy” OR “aquatic rehabilitation”)
Scopus	(TITLE-ABS-KEY (“multiple sclerosis” OR MS) AND TITLE-ABS-KEY (“hydrotherapy” OR “aquatic therapy” OR “aquatic exercise” OR “water-based exercise” OR “water-based therapy” OR “pool therapy” OR “aquatic rehabilitation”))
Web of Science	TS = (“multiple sclerosis” OR MS) AND TS = (“hydrotherapy” OR “aquatic therapy” OR “aquatic exercise” OR “water-based exercise” OR “water-based therapy” OR “pool therapy” OR “aquatic rehabilitation”)
CINAHL	((MH “Multiple Sclerosis+”) OR “multiple sclerosis”) AND ((MH “Hydrotherapy+”) OR “aquatic therapy” OR “aquatic exercise” OR “water-based exercise” OR “water-based therapy” OR “pool therapy” OR “aquatic rehabilitation”)

## Appendix C

**Table A3:** Studies included in each meta-analysis and reasons for exclusion from quantitative synthesis.

Outcome	Include Studies	Excluded Studies	Reason for Exclusion
Fatigue	Castro-Sánchez 2011 [18]; Kargarfard 2012 [33]; Kargarfard 2018 [32]; Razazian 2016 [35]	Bansi 2013 [43,44]	Insufficient quantitative data for pooling
Quality of life	Castro-Sánchez 2011 [18]; Kargarfard 2012 [33]	Bansi 2013 [43,44]	Outcome reporting format not suitable for extraction of mean ± SD values
Mobility and physical function	Aidar 2018 [34]; Kargarfard 2018 [32]	None	Not applicable

Studies were included in quantitative synthesis only when sufficient statistical data (mean and standard deviation) were available for effect size calculation. Abbreviations: SD = standard deviation.
